# Day and night in the subterranean: measuring daily activity patterns of subterranean rodents (*Ctenomys* aff. *knighti*) using bio-logging

**DOI:** 10.1093/conphys/coz044

**Published:** 2019-07-19

**Authors:** Milene G Jannetti, C Loren Buck, Veronica S Valentinuzzi, Gisele A Oda

**Affiliations:** 1 Laboratorio Binacional Argentina-Brasil de Cronobiologia, Departamento de Fisiologia, Instituto de Biociências, Universidade de São Paulo, São Paulo, Brazil; 2 Laboratorio Binacional Argentina-Brasil de Cronobiologia, Centro Regional de Investigaciones Cientificas y Transferencia Tecnológica (CRILAR), Entre Ríos y Mendoza, s/n, Anillaco, La Rioja, Argentina; 3 Department of Biological Sciences, Northern Arizona University, Flagstaff, Arizona, USA

**Keywords:** Activity patterns, subterranean rodents, Ctenomys, nocturnality/diurnality, bio-logging, chronobiology

## Abstract

While most studies of the impacts of climate change have investigated shifts in the spatial distribution of organisms, temporal shifts in the time of activity is another important adjustment made by animals in a changing world. Due to the importance of light and temperature cycles in shaping activity patterns, studies of activity patterns of organisms that inhabit extreme environments with respect to the 24-hour cyclicity of Earth have the potential to provide important insights into the interrelationships among abiotic variables, behaviour and physiology. Our previous laboratory studies with Argentinean tuco-tucos from the Monte desert (*Ctenomys* aff. *knighti*) show that these subterranean rodents display circadian activity/rest rhythms that can be synchronized by artificial light/dark cycles. Direct observations indicate that tuco-tucos emerge mainly for foraging and for removal of soil from their burrows. Here we used bio-logging devices for individual, long-term recording of daily activity/rest (accelerometry) and time on surface (light-loggers) of six tuco-tucos maintained in outdoor semi-natural enclosures. Environmental variables were measured simultaneously. Activity bouts were detected both during day and night but 77% of the highest values happened during the daytime and 47% of them coincided with time on surface. Statistical analyses indicate time of day and temperature as the main environmental factors modulating time on surface. In this context, the total duration that these subterranean animals spent on surface was high during the winter, averaging 3 h per day and time on surface occurred when underground temperature was lowest. Finally, transport of these animals to the indoor laboratory and subsequent assessment of their activity rhythms under constant darkness revealed a switch in the timing of activity. Plasticity of activity timing is not uncommon among desert rodents and may be adaptive in changing environments, such as the desert where this species lives.

## Teaser

Activity patterns of desert subterranean tuco-tucos were measured using light-loggers and accelerometers to understand interrelationships among environment, behavior and physiology. Underground temperatures attain minimal values around noon, favoring diurnal time on surface in winter. Timing of highest overall activity shifts to night-time upon transference from field to lab constant conditions.

## Introduction

Massive changes in climate patterns can compromise organismal fitness and population persistence of many species. Biological systems are dynamic but exhibit stability across time while being responsive to changes in external and internal environments. Characteristically, animals are capable of change either through phenotypic plasticity or microevolutionary processes. While most studies of the impacts of climate change have investigated shifts in the spatial distribution of organisms (Root, 1988; Root *et al.*, 2003; Sinervo *et al.*, 2010), shifts in seasonal phenology (Chevin *et al.*, 2010; Lane *et al.*, 2012; Shen *et al.*, 2015; Williams *et al.*, 2017) or daily timing of activity are other important adjustments that can be made by animals in a changing world (Daan, 1981; Halle and Stenseth, 2000; Kronfeld-Schor and Dayan, 2003; Levy *et al.*, 2018).

The light/dark cycle of Earth imposes a fundamental 24-hour pace that structures the timing of biological activity. In mammals, this is achieved by light/dark entrainment of endogenous, hypothalamic circadian clocks, resulting in rhythmic, 24-hour activity/rest alternations (Rusak, 1982). This endogenously generated rhythm can be profoundly altered by environmental cues, a phenomenon known as ‘masking’ (Aschoff *et al.*, 1982). For example, ambient temperature shapes activity patterns of mammals via direct, acute stimulation or inhibition of behavioural activity (Kenagy, 1973; Melcher *et al.*, 1990; Kenagy *et al.*, 2002a; Rezende *et al.*, 2003; Long *et al.*, 2005). Due to the importance of light and ambient temperature cycles in shaping activity patterns, it is interesting to study their effects on organisms that inhabit extreme environments with respect to the 24-hour cyclicity of Earth. The subterranean environment offers an opportunity for these studies, due to the constant darkness and attenuated temperature cycles of the burrow environment, characteristics thought to diminish the selective pressure of keeping daily activity patterns (Beale *et al.*, 2016).

There are eight lineages of extant subterranean rodents comprised of approximately 146 species (Cook and Lessa, 1998). The most speciose family of subterranean rodents is Ctenomyidae (parvorder Caviomorpha), comprising almost 60 extant species (Cook and Lessa, 1998; Parada *et al.*, 2011) all from the same genus *Ctenomys* sp. (tuco-tucos). This is a relatively understudied group with respect to their activity patterns in the field. Ctenomyids are endemic to South America and are widely distributed from high altitudes to sea level, from moist grass lands to arid deserts (Vassallo, 1998; Lacey *et al.*, 2000). Most of ctenomyids’ diet is composed of aerial parts of plants; therefore, their foraging occurs primarily on the surface (Lacey *et al.*, 2000). This may explain why tuco-tucos have maintained functional eyes of moderate size, in contrast to spalacids and bathyergids of Africa and Asia (Borghi *et al.*, 2002).

Direct observations of individual tuco-tucos from northern Monte desert Argentina (*Ctenomys aff. knighti*), indicate that they emerge to the surface mainly for foraging and soil removal associated with tunnelling (Tomotani *et al.*, 2012). Automated recordings of light-exposure using light-loggers confirmed that tuco-tucos emerge to the surface on a daily basis (Flôres *et al.*, 2016). Standard chronobiological lab protocols showed that their circadian clocks are functional and entrained by light/dark cycles (Valentinuzzi *et al.*, 2009; Flôres *et al.*, 2013) and through computer simulations it is clear that the unique light exposure patterns of tuco-tucos observed in the field are sufficient to synchronize their biological clocks (Tomotani *et al.*, 2012; Flôres *et al.*, 2016).

Several studies in non-subterranean rodents have shown that air and operative temperatures play an important role in determining whether the animal stays on the surface or retreats to underground burrows for thermal shelter (Melcher *et al*., 1990; Kenagy *et al*., 2002a; Rezende *et al*., 2003; Long *et al*., 2005). Conversely, subterranean tuco-tucos spend most of their time belowground, only emerging to the surface for brief episodes. It is not clear, in this scenario, whether air temperature plays a similar role in promoting surface emergence or how underground temperature influences this decision.

We hypothesized that an interplay between these two temperatures would have masking effects on the timing of tuco-tuco emergence to the surface. To test this main objective, we outfitted tuco-tucos with light-loggers and simultaneously measured air, operative and underground temperatures and wind velocity.

We additionally quantified phase, amplitude and duration of overall daily activity by deploying miniature accelerometers to four of the animals with light-loggers. This second objective was motivated by the fact that the diurnal surface emergence of tuco-tucos in the field contrasts with the nocturnal wheel-running activity under laboratory conditions (Valentinuzzi *et al*., 2009; Tachinardi *et al*., 2014). This apparent difference in phase of activity between the laboratory and field could have been due to different methods of assessing activity (i.e. wheel running in the laboratory and light-loggers in the field); thus, our current study is the first to test activity timing in both the laboratory and field employing a single method (accelerometry) to quantify activity.

## Methods

### Study population

Tuco-tucos *Ctenomys aff. knighti* occur in Anillaco and surroundings, in La Rioja, Argentina (28°48’S; 66°56’W; 1445 m). This arid to semiarid area is located in the Monte desert with average daytime temperatures ranging between 18°C in winter and 28°C in summer and average nighttime temperatures between 9°C in winter and 18°C in summer [2006–16 AIRX3STM v006 dataset, obtained from Giovanni online data system, developed and maintained by the NASA GES DISC (Acker and Leptoukh, 2007)]. Average annual precipitation is variable, ranging between 100 and 450 mm. Precipitation events are generally limited to summer and droughts can last up to 7 months (Abraham *et al.*, 2009). The soil is sandy and the predominant vegetation in these regions is a shrubby steppe, with characteristic Zygophyllaceae, Fabaceae and Cactaceae flora (Fracchia *et al.*, 2011; Aranda-Rickert, 2014).

Seventeen adult tuco-tucos *Ctenomys aff. knighti* were live trapped in Anillaco from 2016 to 2017 using custom made traps (Tomotani *et al.*, 2012). Captured animals were hand carried in traps to the adjacent laboratory inside the *Centro Regional de Investigaciones Científicas y Transferencia Tecnológica*, located in Anillaco. Tuco-tucos were weighed (CSseries, OHAUS, ± 1 g precision) and placed individually into acrylic cages with wire tops (53 × 29 × 27 cm) for an average of 1 month. Individuals were uniquely marked with a subcutaneously injected microchip (Allflex®, Brazil). Cages were kept in a room with constant temperature 24 ± 2°C, natural lighting and minimal noise. Animals were fed once a day at arbitrary hours. Food consisted of carrots, sweet potatoes, oatmeal, sunflower seed and rodent pellets. Tuco-tucos do not drink free water (Buffenstein, 2000).

All procedures of this work followed the guidelines established by the *American Society of Mammalogists for animal care and handling* (Sikes, 2016), and were approved by the Comissão de Ética no Uso de Animais from *Instituto de Biociências—USP* (n° 273/2016), and were authorized by the *Dirección General de Ambiente y Desarrollo Sustentable—Secretaría de Ambiente del Ministério de Producción u Desarrollo Local*, La Rioja, Argentina (n° 028/2010 and 062/2008).

### Arenas and environmental measurements

Tuco-tucos were maintained in one of three semi-natural outdoor enclosures (arenas) containing native vegetation. The arenas are adjacent to one another and surrounded by wire fencing above ground (arena 1, 10 × 6 × 1.5 m or arenas 2 and 3, 12 × 6 × 1.5 m) and concrete block 1 m below ground to inhibit escape of the tuco-tucos. A mesh covered each arena to prevent aerial predation. Only one animal at a time was kept inside each arena. No food was provided during the experiments; thus, animals were reliant upon foraging on natural vegetation.

A meteorological station (HOBO®, Onset, USA), 1 m from the arenas, provided hourly records of air temperature (T_air_) (at 1.5 m high) and wind velocity (at 2.1 m height). Subsurface temperatures were measured and recorded each hour (HOBO®, Onset, USA; accuracy ±0.53°C from 0°C to 50°C) at 20 (T_und_), 40 and 60 cm underground using sensors buried in sand. Reported average burrow depths of *Ctenomys* sp. range from 13 cm to 45 cm (Altuna *et al*., 1999; Antinuchi and Busch, 1992). Operative temperature (T_e)_ was measured and recorded each hour (HOBO®, Onset, USA; accuracy ±0.53°C from 0°C to 50°C) by a sensor wrapped in copper and inserted inside a taxidermied animal (‘mannequin’) (Bennett *et al.*, 1984; Kenagy *et al.*, 2002a) filled with cotton. The cotton filled mount was validated against a dead animal (Supplementary Fig. 1). The mannequin was exposed to direct sunlight in the soil surface of a fenced area of an arena. Continuous exposure to the elements and concomitant degradation required that mannequins be exchanged every 6 months. In 2015, 2016 and 2017, we obtained T_e_ data for 17%, 66% and 81% of the year, respectively. Environmental variables from the meteorological station and underground sensors were recorded without interruption from 2015 to 2017. Photoperiod, as well as civil twilight times (day and night onsets), were obtained from data available online (Time and Date AS, 2018).

### Measurement of activity/rest rhythm

Bio-loggers were used to assess light exposure (i.e. time on surface) and gross motor activity of tuco-tucos in the arenas. Both loggers were affixed to a collar made of a cable tie inserted through pliable silicon tubing (Williams *et al.*, 2014). Seventeen animals were affixed with bio-loggers, 11 of them with both accelerometer and light-logger and 6 with only a light-logger. We obtained records for six individuals (five females 141 g ± 23 g and one male 191 g). Four of these (three females and one male) had both a light logger and an accelerometer and the remaining two only had light loggers. One of our original 17 animals died of unknown causes and the remaining 10 individuals either escaped or were lost to predation. The loss of animals to predation and escape was both surprising and unfortunate given the construction of the arenas that included a 1-m buried wall and fencing around and across the tops of the arenas. Clearly, a 1-m buried wall is not always sufficient to contain tuco-tucos and wire mesh fencing is not adequate to block the entrance of some predators (e.g. snakes). In this context, light exposure data of six individuals recorded for 7 days in July and August of 2015, five of them from Flôres *et al*. (2016), were added to our analyses of time on surface, to increase sample size. The same environmental measurements described above were taken during all of these additional recordings, except for T_e_. Since we had T_e_ data for only 17% of the year of 2015, only four of the additional individuals from Flôres et al. (2016) were used when verifying association between time on surface and operative temperature.

Time on surface was deduced from light exposure, sampled every 1 minute and only the maximum sample value recorded within each 5 minutes by light loggers (15 × 6 × 6 mm; 0.65 g; W 65, Migrate Technology, UK). Measurement range of light loggers is 1–19 000 lux and resolution is of 249 discrete levels, with no sensitivity to detect moonlight. Gross motor activity was recorded every 1-second in three spatial axes (XYZ) via accelerometry (23 × 12 × 10 mm; 2 g; Axy-3, TechnoSmart, Italy) with measurement range of ±4 G-forces.

### Experimental protocol

This study was conducted across 2 years (2016–17) during the months of April–August. The six animals from which we have data were released individually inside the arenas and were re-captured to recover devices. Deployment duration ranged from 8 to 68 days, because the duration was adjusted throughout the experiment, considering that increased deployment durations also increased chance of death, predation or escape. Individual animals were only tested in the arenas one time. Re-captured animals were taken to the laboratory where devices were removed and data were downloaded. Animal #221 took part on a parallel experiment after its recapture. Therefore, three of the four animals with accelerometers were kept with their sensors upon arrival and then transferred to cages placed inside light-tight ventilated boxes, under constant darkness (DD) and constant temperature (24°C ± 2°C) conditions. Food was provided once every two nights at arbitrary times. Activity of these animals was recorded by accelerometers over at least 10 days.

### Analysis

All mathematical operations and statistical analyses were performed using R software (R Core Team, 2018). Light-logger recordings with values ≥2 lux were considered episodes on surface. Acceleration data were collected from the three orthogonal axes and used to calculate the overall dynamic body acceleration (ODBA) as described in Williams *et al.* (2016). This method consists of subtracting the moving average from the data of each axis, using a 10-second time window. ODBA sums the resulting absolute values. Activity episodes were defined by episodes where ODBA values were higher than the arithmetic mean of all values from that animal, which was ground truthed against laboratory observations. All ODBA values below the arithmetic mean were considered episodes of ‘low body movement’, equivalent to rest. Additionally, high activity episodes for each animal were defined as episodes of body movement higher than half of the maximum value from that animal, which represents the majority of the activity bouts that can be visually observed in the actograms and supposedly corresponds to energy demanding activities.

Time on surface and gross activity data were visualized in actograms made with El Temps software (Díez-Noguera, 2019). Data from both the light and activity loggers were superposed to qualitatively evaluate the temporal association between the two variables.

For the four individuals from which we have information on both acceleration and light exposure, we calculated the percentage of time, relative to 24 hours, in which activity episodes occurred (‘activity’ vs. '’rest’). From total daily activity time, we also calculated the percentage of ‘diurnal’ vs. ‘nocturnal’ activity, correcting for the day length of each season, using the following formula modified from Halle and Stenseth (2000)

$$ P=\frac{\frac{\sum {c}_L}{h_L}}{\frac{\sum {c}_L}{h_L}+\frac{\sum {c}_D}{h_D}}\times 100, $$

where P is the percentage of diurnal activity, $\sum {c}_L$ and $\sum {c}_D$ are the sum of activity records obtained during the day and night, respectively and ${h}_L$ and ${h}_D$ are the duration of day and night (civil twilight times). As noted above, the light logger only detects time on surface that occurs in daylight. Therefore, we also calculated the percentage of diurnal activity that occurred on the surface (‘surface’ vs. ‘subterranean’). High activity bouts (body movements higher than half of the maximum value of each animal) were also quantified. We obtained percentage of high activity bouts that happened during daylight hours and those that happened when animals were on the surface. For each of the three animals transferred to the lab, the average value of overall activity in the enclosures and in the lab were compared in order to quantitatively measure the difference between them. The resulting proportions for each animal were averaged.

**Figure 1:**
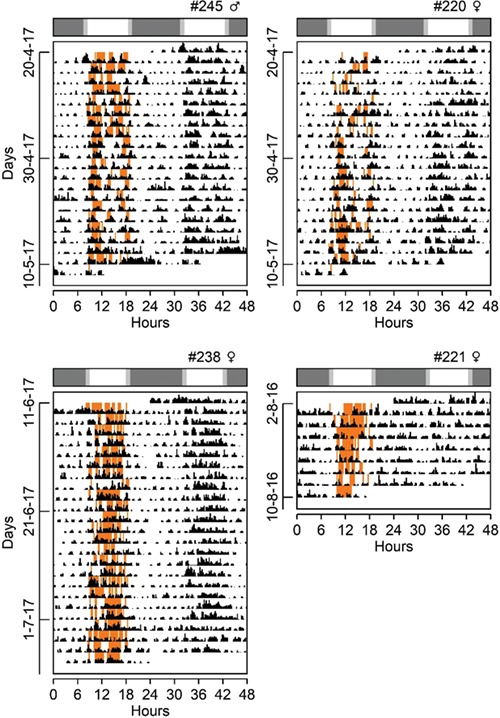
Double-plotted actogram of daily rhythms of gross motor activity and diurnal time on surface of tuco-tucos kept inside arenas in the field in Anillaco, La Rioja, Argentina, from April to August of 2016 and 2017. Orange marks: time on surface during daylight hours. Black marks: gross motor activity measured with accelerometers. Bar above actograms: natural photoperiod at which each recording started (white: day; grey: night). Top right of each actogram: animal identification number and sex.

**Figure 2:**
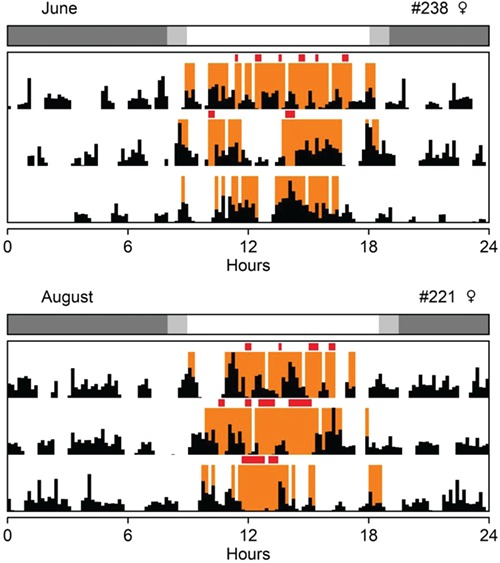
Three-day actogram of individuals #238 and #221 inside arenas in the field. Orange marks: time on surface during daylight hours. Black marks: gross motor activity measured with accelerometers. Red marks: aboveground episodes with low body movement. Bar above actograms: natural photoperiod at which each recording started (white: day; grey: night). Measurements were made on August 2016 (#221) and June 2017 (#238).

Gathering data from light loggers of the six animals (2016–17) and the six additional animals recorded in 2015, we verified possible differences between the amount of time on surface of males and females with unequal variance t-test (Ruxton, 2006). We used a generalized linear mixed model (‘glmmTMB’ function) to evaluate the degree of masking on time on surface by environmental variables (Zuur *et al.*, 2009). Light logger data from 10 animals were used in this analysis, since T_e_ data was not collected simultaneously for two animals recorded in 2015. Proportion of surface episodes within 3-hour intervals were used as a response variable. This was calculated as the percentage of episodes classified as aboveground (light-logger values equal to or higher than 2 lux) out of a total of 36 recordings (3-hour interval). Explanatory variables in our model were T_e_, T_und_, wind speed (Wind) and time of day as a categorical variable, divided in 3-hour blocks (Hour), which included the following categories: ‘A’ for hours 09:00, 10:00 and 11:00; ‘B’ for 12:00, 13:00 and 14:00; ‘C’ for 15:00, 16:00 and 17:00; and ‘D’ for 18:00, 19:00 and 20:00, considering that environmental measurements were made each hour. Animal identification number (ID) was inserted as a random effect variable. Possible linear correlations between independent variables were verified in linear models, considering correlations significant when |R| ≥ 0.5. Variance inflation factor (VIF) was also calculated to check for collinearity between independent variables included in each model. GVIF values presented for Hour were corrected for degrees of freedom according to Fox and Monette (1992) and squared for comparison with VIF values of other variables. Final model selection was based on absence of correlated independent variables and use of AIC value, selecting the model with lowest AIC.

We calculated and summarized the average duration of time on surface for each hour of the day. To every animal’s record, we obtained a curve, calculating the average duration of time on surface for each time of day. All animals’ mean curves were then summarized as an average curve (*n* = 12). Similar steps were done to obtain duration of time on surface for each interval of T_e_ (*n* = 10) and each interval of T_und_ (*n* = 12). All data collected at night were excluded from these calculations since light-loggers do not detect time on surface when it is dark. Pearson’s linear correlation coefficient was calculated for the average points of surface episodes × T_e_ and surface episodes × T_und_ final curves. Finally, data of duration of surface episodes per hour and per temperature intervals (T_e_ or T_und_) were visualized simultaneously in three axes figures.

## Results

During the April to August (winter) study period, T_e_ daily range was 28.9°C on average. The maximum T_e_ was 50.2°C at 13:00 in August 2015 and T_e_ minimum was −11.8°C at 08:00 in July 2017. Average daily range of T_und_ was 3.7°C. T_und_ maximum was 23.8°C at 00:00 in May 2015 and T_und_ minimum was 5.2°C at 15:00 in June 2016. The lowest values of T_und_ at 20 cm deep occurred regularly near noon, when T_e_ values were the highest. When T_und_ was compared to deeper measurements, the latter presented a more delayed and flatter variation profile (Supplementary Fig. 2). In La Rioja, maximum day length is 14.03 hours at summer solstice and minimum day length is 10.25 hours at winter solstice.

Accelerometer data revealed that, in addition to activity during the day, *Ctenomys aff. knighti* are also active during the night (Fig. 1). However, on average, 77% of high activity bouts occurred during the daylight hours. Additionally, 47% of these high activity bouts corresponded to when the animals were on the surface. Interestingly, two of the four individuals outfitted with both light and acceleration loggers displayed episodes of low body movement (ODBA below the arithmetic mean) while on the surface (Fig. 2).

Considering all activity episodes (ODBA values above the arithmetic mean), animals were active on average 14.47 hours (60%) of the day, and three of the four individuals expressed >60% (8 hours) of their activity during daylight hours. There was an interindividual standard variation of 1 hour (8.6%) in the time that animals spent on the surface (Table 1). Considering all light logger recordings (*n* = 12), the amount of time on surface did not differ between males and females (unequal variance t-test *P* = 0.9) (Supplementary Table 1).

**Table 1:** Quantification of daily activity duration of the four tuco-tucos with both activity and light loggers, when kept inside arenas in the field

Animal number	D	Activity (%)	Rest (%)	Diurnal (%)	Nocturnal (%)	Surface (%)	Underground (%)
245	20	61.8	38.2	64.7	35.3	30.9	69.1
220	20	54.1	45.9	64.1	35.9	18.2	81.8
238	24	60.5	39.5	66.7	33.3	36.2	63.8
221	8	64.9	35.1	44.9	55.1	37.7	62.3
Average	18 ± 7	60.3 ± 4.5	39.7 ± 4.5	60.1 ± 10.2	39.9 ± 10.2	30.8 ± 8.9	69.2 ± 8.9

Shown is the percentage of gross motor activity vs. rest (body movements below average value for each individual) related to the 24 hours of the day; percentage of diurnal vs. nocturnal activity related to total activity time (corrected for day length, using formula described in ‘Methods’); and percentage of surface vs. underground activity related to total diurnal activity. D: number of recording days included in the calculations for each animal.

**Figure 3:**
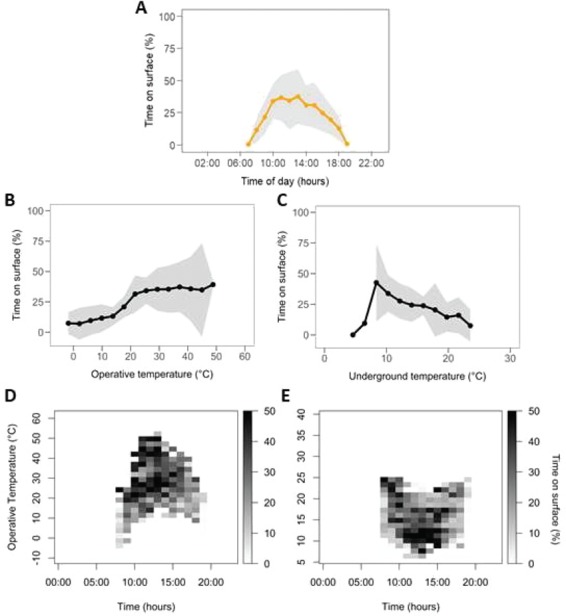
Average percentage of recordings of animals on surface when kept inside arenas in the field, from April to August of 2015–17, at each time of day (**A**), each operative temperature interval (**B**) or each underground temperature (at 20 cm) interval (**C**). Shaded area: standard deviation between individuals. (A) Sample size: 12 individuals; (B) Sample size for each point respectively: 1, 5, 10, 10, 10, 10, 10, 10, 10, 10, 9, 8, 8, 5, 2; (C) Sample size for each point respectively: 1, 1, 3, 7, 7, 8, 7, 9, 6, 5, 3. Simultaneous effect of time of day and environmental temperature is shown in (D) for operative temperature and (E) for underground temperature. Darker areas indicate intervals of temperature and time of day when percentage time on surface was higher.

On average, time on surface was concentrated around noon (Fig. 3). Despite the high standard deviation of average values of time on surface, this variable increased with increasing values of T_e_ (Pearson correlation coefficient for average points: R^2^ = 0.88, *P* < 0.001). Standard deviation of time on surface increased when T_e_ > 25°C. The time tuco-tucos spent on surface increased when underground temperature at 20 cm deep decreased. There was one exception to this pattern where, in a particularly cold, snowing day, individual (#193) was on the surface for an average 5.6 minutes (10% of 1 hour) when T_und_ was 6.5°C and remained underground (0% time on surface) when T_und_ was 4.6°C (Pearson correlation coefficient for average points: R^2^ = 0.20, *P* = 0.10 considering these occurrences and R^2^ = 0.92, *P* < 0.001 without these occurrences). When both time of day and T_e_ or T_und_ effects are considered together, it is evident that while time on surface between 10:00 and 15:00 occurs across a wide range of T_e_, within this timespan, episodes on surface occur mostly when T_und_ values are limited to 7–12°C.

**Figure 4:**
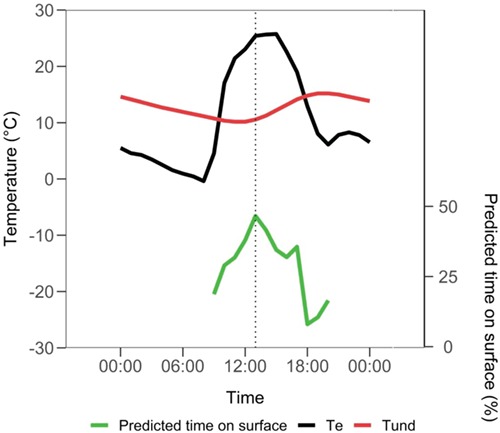
Predicted relationship of time on surface with T_e_ and T_und_ in tuco-tucos, based on the generalized linear model. This prediction was constructed with time of day, T_e_, T_und_ and Wind values of an arbitrary day (15 July 2016), in Anillaco, La Rioja, Argentina. Green line: predicted time on surface; black line: T_e_; red line: Tund. Wind speed variation was omitted for better clarity.

The final generalized linear mixed model included T_und_, T_e_, Hour and Wind, which explained almost 60% of the duration of time on surface (Table 2), accounting for the random effect of animal’s ID (contribution of 7.7%). Models with both T_e_ and T_air_ were not considered since linear correlation between these variables was 67% (*P* < 0.001) and these models resulted in VIF of 5.6 or higher for T_e_. The second highest linear correlation value was between T_e_ and Hour (45%, *P* < 0.001). Considered separately, T_e_ explained 31% of time on surface (12% attributed to random effect); however, due to its correlation with Hour being close to the threshold of 50% previously established, high R^2^ values of T_e_ + Hour models must be viewed with caution (in T_e_ and Hour model, GVIF for Hour: 1.3; VIF for T_e_: 2.4). Meanwhile, when T_und_ is considered separately, this model’s R^2^ is 16% (5% attributed to random effect). Furthermore, the model with T_und_ and Hour (GVIF for Hour: 1.1; VIF for T_und_: 1.2) received an AIC score 30 units lower than that including T_e_ and Hour, indicating a higher contribution of T_und_ than T_e_ to explain time on surface. This is observed in the projection of the model (Fig. 4), where time on surface increases around 12:00, when T_und_ is minimal and T_e_ is maximal.

**Figure 5:**
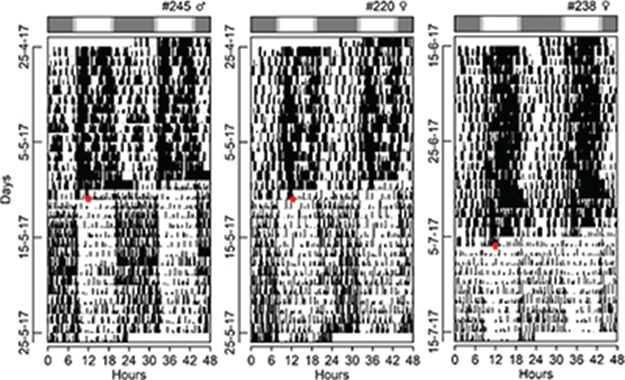
Double-plotted actogram of daily rhythms of gross motor activity of tuco-tucos kept inside arenas in the field and then released into constant darkness in the lab. Black marks: gross motor activity measured with accelerometers. Bar above actograms: natural photoperiod at which each recording started in the field (white: day; grey: night). Red arrows indicate day and time of transference. Top right of each actogram: animal identification number and sex. Because daily activity levels were lower in the lab than in the field, the upper limit of activity used in this actogram was lower than that used in Fig. 1, to allow visualization of activity/rest throughout the entire field/lab transition in a single actogram.

The three individuals outfitted with accelerometers and transferred to laboratory under DD conditions also displayed activity bouts during both day and night in the lab. However, while in the outdoor arenas these animals expressed 77% of their high activity bouts during daytime, this proportion decreased to just 15% upon transfer to the lab (i.e. 85% of their high activity bouts happened during external nighttime hours) (Fig. 5). Finally, comparing average field and lab levels for each individual, overall daily activity levels were on average 1.6 times higher in the field than in the lab.

## Discussion

Studies of activity patterns in the field and laboratory allow us to understand ecological aspects as well as endogenous mechanisms of biological timing and its plasticity (Marques and Waterhouse, 2004). However, recording of activity in the field constitutes a logistical challenge (Halle and Stenseth, 2000; Dominoni *et al.*, 2017), particularly for subterranean rodents, with varying degrees of underground and aboveground activity. While most studies of activity patterns in free-living subterranean/fossorial mammals have been conducted with mole rats from Africa and Asia (Nevo *et al.*, 1982; Ben-Shlomo *et al.*, 1995; Lovegrove and Muir, 1996; Riccio and Goldman, 2000; Oosthuizen *et al.*, 2003; Sklíba *et al.*, 2007; Vlasatá *et al.*, 2017), caviomorph subterranean/fossorial species from South America have also added important insights to this comparative study (Kenagy *et al.*, 2002a, 2002b; Rezende *et al.*, 2003; Tomotani *et al.*, 2012; Estevan *et al.*, 2016). In particular, the influence of ambient temperature on the surface activity of subterranean/fossorial rodents has been well studied in the South American degu (Kenagy *et al*., 2002a, 2002b) and coruro (Rezende *et al*., 2003).

Several studies with epigeous species have analysed the correlation between burrow retreats and ambient temperatures, indicating that burrows serve as thermal refugia for these species that live mostly aboveground (Melcher *et al*., 1990; Long *et al*., 2005; Fick *et al*., 2009). This is particularly important for desert rodents that can unload excess heat inside cool burrows, saving evaporative water in thermoregulation (Bartholomew 1964; Chappell and Bartholomew, 1981; Bennett *et al*., 1984). Conversely, underground temperature plays an important role for subterranean tuco-tucos, in the opposite movement of emergence to the surface, in winter. Across most of day in winter, underground temperature (14.3°C on average) is below the thermoneutral zone of tuco-tucos (23–33°C) (Tachinardi *et al.*, 2017) and a 24-hour variation is detectable at 20-cm depth with amplitude 3.7°C. Interestingly, temperature maxima occur around midnight and minima around noon, coinciding with the highest operative temperatures aboveground. Thus, emergence to the surface during the day is favoured for thermoregulatory reasons, which explains the high total time subterranean tuco-tucos spend on the surface (24% of day time, 3 hours per day on average) in this season. Finally, a higher contribution of T_und_ than T_e_ to explain time on surface is further observed in the projection of our generalized linear mixed model (Fig. 4). Although time on surface increases around 12:00 when T_und_ is minimal and T_e_ is maximal, it starts to decrease while T_e_ remains high, from 13:00 to 15:00, along with an increasing T_und_.

**Table 2:** Comparison between generalized linear mixed models performed with environmental variables recorded, to explain variability of time on surface every 3 hours

Fixed effect variables	AIC	R^2^ (%)	χ^2^
**T_und_, T_e_, Hour, Wind**	**3600.3**	**59.9**	**512.6**
T_und_, T_e_, Hour	3635.7	55.6	473.2
T_und_, Tair, Hour	3683.7	57.5	425.2
T_und_, Hour	3691.2	53.1	421.7
T_e_, Hour	3721.4	59.4	391.5
Hour	3829.0	54.3	265.9
T_e_	4006.0	31.1	84.9
T_und_	4049.8	16.3	41.1
Wind	4067.5	11.3	23.4
Tair	4069.2	12.8	21.7

R^2^ values consider the random effect variable, animal ID. Final model, in bold, is further discussed in the text.

Number of observations: 633 from 10 animals. All models above were significant with P < 0.001.

Interestingly, combined light-logger and accelerometer data in winter revealed several incidences of low activity while animals were on the surface (Fig. 2). This may be indicative of basking, which would be in accordance with the above association between time on surface and thermoregulation. Basking has been traditionally associated with ectotherms (Bogert, 1949) but recent findings have established their role in small rodents during early mornings, rewarming from daily torpor (Geiser *et al.*, 2002; Warnecke *et al.*, 2008). Basking at random times has been seen less often, but has been identified in subterranean rodents such as the African ice rat (*Otomys sloggetti robertsi*) (Schwaibold and Pillay, 2006) and African mole rat (*Tachyoryctes microcephalus*) (Vlasatá *et al*., 2017). We hypothesize that tuco-tucos are also basking during those times on the surface without movement. Our previous observational studies indicated that tuco-tucos do not wander on the surface for foraging purposes; but rather, they only leave the burrow for very few seconds to acquire vegetation and immediately return (Tomotani *et al*., 2012). The only activity they perform with their whole body exposed for long durations (minutes to an hour) is soil removal, an activity that coincides with their highest locomotor activity aboveground (Tomotani *et al*., 2012). However, they can stay up to 1 hour with only their heads out of the burrow entrance without doing any noticeable movement (M Jannetti, personal observation). While it also looks like a vigilant state, we hypothesize that this very common, immobile posture, with only the heads out of the burrow could be basking. If future studies using body temperature sensors confirm this hypothesis, we can conclude that thermoregulatory basking is another important, winter behaviour of tuco-tucos on the surface, besides foraging and soil removal.

Based on the considerable amount of time tuco-tucos spend on the surface (Flôres *et al*., 2016) and on the nature of their observed surface behaviours (Tomotani *et al*., 2012), we had concluded previously that they are diurnal in the field. However, we had no knowledge of their activity timing and behaviours underground. The combination of our light-logger and accelerometer data revealed that highest activity levels do in fact occur when tuco-tucos are on the surface. However, they also revealed that lower amplitude activity occurs underground during both the day and the night (Fig. 1). Because accelerometers can be used in both the field and lab, we then transferred three of the four individuals to the laboratory for continued monitoring of their activity timing under constant dark conditions. This was the first time we tracked a single activity parameter, the locomotor activity level from ODBA from the same individuals under both captive and field conditions. Activity during both day and night was again detected in lab condition but, similar to our previous experiments using light-loggers in the field and wheel-running in the lab, accelerometry data confirmed the shift in highest activity time between field and lab conditions (Fig. 5). The night-time activity displayed by tuco-tucos under constant darkness in the lab represents the circadian clock controlled component (Hut *et al.*, 2011; Tomotani *et al.*, 2012) while the timing of field activity is defined downstream the clock (van der Vinne *et al.*, 2014). While the former depends mostly on the stable light/dark entrainment of the circadian clock (Flôres *et al.*, 2016), the latter is plastic and susceptible to prevailing environmental conditions, with higher activity levels during the day associated to economy in thermoregulatory costs (van der Vinne *et al.*, 2014, 2015). Finally, we verified that overall activity levels in the field is on average 1.6 times greater than in the lab, a discrepancy that has always been assumed to explain the temporal niche switches between field and lab conditions in rodents (Hut *et al*., 2011; van der Vinne *et al*., 2014).

Although higher levels of activity are concentrated during the day while in the field, accelerometry clearly revealed that tuco-tucos also display significant activity at night (40%) during the winter (Fig. 1). A similar pattern, but with a more evenly distributed activity bouts throughout day and night, has been observed in several primate species in the field (Erkert and Cramer, 2006). Erkert and Cramer (2006) have experimentally shown the circadian component behind this arrythmic pattern and suggest that this may represent a transitory stage on the way from a nocturnal to a diurnal lifestyle. This is an interesting proposition, given the common nocturnal ancestry of mammals (Gerkema *et al.*, 2013) and that the capacity of an organism to be active in either day or night, depending on prevailing environmental conditions, is viewed as adaptive in a changing environment.

The climate of South American deserts is changing, particularly with respect to increase in temperature and decrease in precipitation (Boulanger *et al.*, 2006; Labraga and Villalba, 2009). Desertification has become an enormous ecological problem, caused mainly by vegetation destruction, soil erosion and lack of water. In the desert of La Rioja province, Argentina, tuco-tucos play an important role in their environments, due to their burrowing habits, constantly altering the soils, dispersing diverse organic materials (Malizia *et al.*, 2000; Lara *et al.*, 2007), mycorrhizal fungi and endophytes, which form symbiotic relationships with the indigenous plant species (Fracchia *et al*., 2011). However, their biology is poorly known and they live under constant human pressure since the local community encourages their extermination.

Climate projections suggest that vegetation cover and water availability will decrease under future climate scenarios (Therburg *et al*., 2019); thus, limiting the ability of animals to remain active during the hotter day hours. The 24-hour time axis is an ecological resource that some animals can exploit to buffer impacts of climate change (Levy *et al*., 2018). Plasticity of activity timing is seen in several other desert rodents (Levy *et al*., 2007) and may be adaptive in changing environments. It is imperative to understand the capacity of animals to switch temporal niches, much as it is important to understand their capacity to migrate, when making predictions of how organisms will cope with these new climate-related challenges. Our data on biological timing in tuco-tucos indicate a high degree of phenotypic plasticity in activity timing as evidenced by their capacity for temporal niche switching and both day and night activity.

## Supplementary Material

Supplementary_Figure_1

Supplementary_Figure_2

Supplementary_Table_1_c
