# Supplementary material for: Day and night in the subterranean: measuring daily activity patterns of subterranean rodents (Ctenomys aff. knighti) using bio-logging
Source: Conserv Physiol. 2019 Jul 19;7(1):coz044. doi: 10.1093/conphys/coz044 (PMC6640163; doi:10.1093/conphys/coz044)
Supplement: Supplementary_Figure_1 [file supplementary_figure_1.docx]

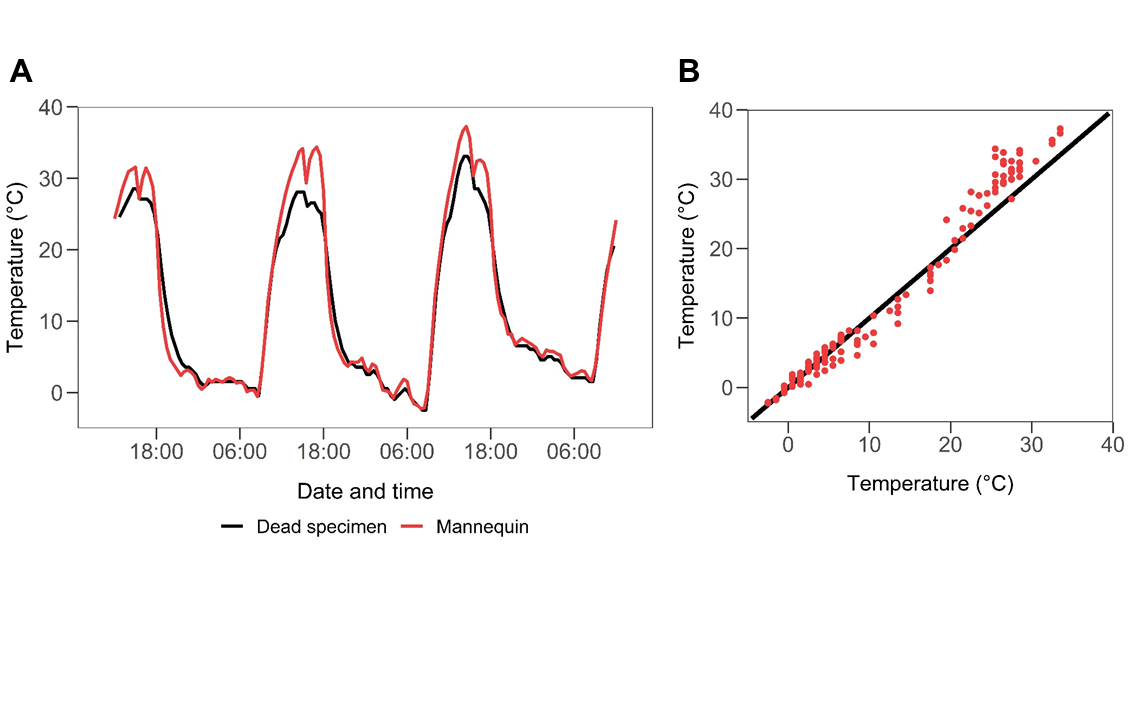


**Supplementary Figure 1. Comparison between operative temperature measured with a cotton filled mount and temperature inside a dead specimen of tuco-tuco, in time-series format (A) and using temperature of dead specimen as the reference line (B). Measurements were made simultaneously during 3 days in July 2017 inside a semi-natural enclosure. Recordings of T_e_ are represented in red and recordings of dead specimen, in black.**
