# Supplementary material for: Day and night in the subterranean: measuring daily activity patterns of subterranean rodents (Ctenomys aff. knighti) using bio-logging
Source: Conserv Physiol. 2019 Jul 19;7(1):coz044. doi: 10.1093/conphys/coz044 (PMC6640163; doi:10.1093/conphys/coz044)
Supplement: Supplementary_Figure_2 [file supplementary_figure_2.docx]

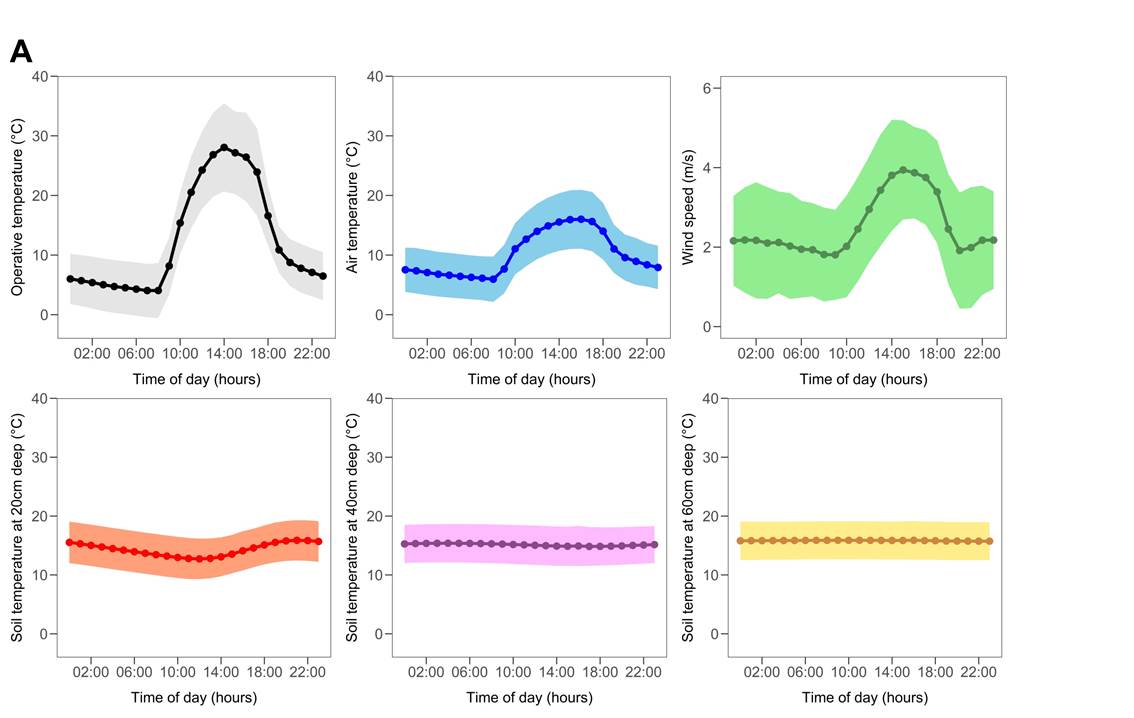

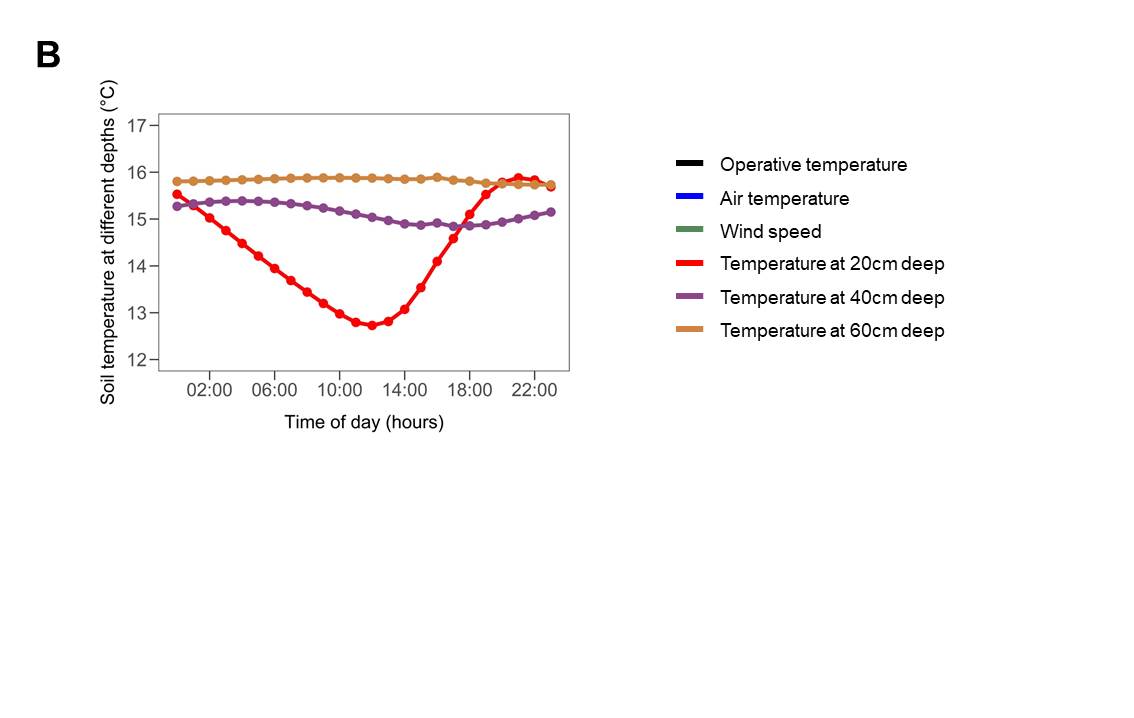


**Supplementary Figure 2. Average daily variation of environmental variables measured at 1m from experimental arenas from April 20^th^ to August 10^th^, 2016 and 2017, in Anillaco, La Rioja, Argentina. A: Daily variation of each variable, separately. Points: average values of temperature or wind speed obtained for each hour of the day; shaded area: standard deviation. B: Comparison of average daily variation of soil temperatures at 20(T_und_), 40 and 60cm deep, in a higher scale.**
