# Supplementary material for: Day and night in the subterranean: measuring daily activity patterns of subterranean rodents (Ctenomys aff. knighti) using bio-logging
Source: Conserv Physiol. 2019 Jul 19;7(1):coz044. doi: 10.1093/conphys/coz044 (PMC6640163; doi:10.1093/conphys/coz044)
Supplement: Supplementary_Table_1_c [file supplementary_table_1_c.docx]

Supplementary Table 1. Proportion of time that tuco-tucos with light loggers spent on the surface relative to daylight hours when kept inside arenas in the field. D: number of recording days for each animal. The percentage calculation accounted for differences in day length.

| Animal number and sex | D | Time on surface (%) | Time on surface (hours) | Average daylight hours |
| --- | --- | --- | --- | --- |
| 177 ♂ | 57 | 19.6 | 2.3 | 11.6 |
| 180 ♀ | 8 | 17.9 | 2.1 | 11.5 |
| 183 ♂ | 9 | 36.0 | 4.2 | 11.7 |
| 184 ♂ | 13 | 18.2 | 2.1 | 11.7 |
| 185 ♀ | 8 | 35.5 | 4.1 | 11.5 |
| 188 ♂ | 9 | 9.2 | 1.0 | 11.4 |
| 193 ♀ | 68 | 24.4 | 2.8 | 11.6 |
| 220 ♀ | 20 | 13.1 | 1.6 | 11.9 |
| 221 ♀ | 8 | 32.0 | 3.8 | 11.8 |
| 222 ♀ | 12 | 26.0 | 3.0 | 11.7 |
| 238 ♀ | 24 | 30.9 | 3.5 | 11.3 |
| 245 ♂ | 20 | 24.7 | 3.0 | 11.9 |
| Average | 21 ± 20 | 24 ± 8.6 | 2.8 ± 1 | 11.6 ± 0.2 |
